# Patterns and determinants of COVID-19 mortality in Bangladesh: insights from three health and demographic surveillance systems across diverse socio-environmental settings

**DOI:** 10.1186/s12963-025-00448-z

**Published:** 2026-06-04

**Authors:** Md Mehedi Hasan, Susmita Das, Chodziwadziwa Whiteson Kabudula, Srizan Chowdhury, Konok Akter, Jean Juste Harrisson Bashingwa, Sayed Saidul Alam, M. Zahirul Haq, Munirul Alam Bhuiyan, M Moinuddin Haider, Abdur Razzaque, Beth A. Tippett Barr, Stephen Tollman, Syed Manzoor Ahmed Hanifi

**Affiliations:** 1 Health Systems and Population Studies Division, International Centre for Diarrhoeal Disease Research, Bangladesh (icddr,b), Dhaka, Bangladesh; 2 SAMRC/Wits Rural Public Health and Health Transitions Research Unit (Agincourt), School of Public Health, Faculty of Health Sciences, University of the Witwatersrand, Johannesburg, South Africa; 3 Nyanja Health Research Institute, Salima, Malawi; 4 Global Health and Migration Unit, Department of Women’s and Children’s Health, Uppsala University, Uppsala, Sweden

**Keywords:** COVID-19, Mortality, HDSS, Matlab, Chakaria, Urban slum, Bangladesh

## Abstract

**Background:**

The COVID-19 pandemic has severely affected health and well-being worldwide. While most countries have reported excess mortality associated with COVID-19, little is known about individual and social factors associated with COVID-19 and non-COVID-19 mortality in Bangladesh. This study addresses that gap by investigating the mortality rates from COVID-19 and other causes during the pandemic years (2020–2021), as well as their associated socio-demographic determinants using longitudinal population data.

**Methods:**

From 2020 to 2021, data were collected on 573,433 individuals residing in three HDSS areas in Bangladesh: Matlab (rural), Chakaria (coastal), and the slums of Dhaka (urban). Probable causes of death were determined by medical personnel using the WHO 2016 verbal autopsy (VA) tool supplemented with a COVID-19 module. Deaths were classified as COVID-19 or non-COVID-19 using the International Classification of Diseases and Related Health Problems, tenth revision (ICD-10). Factors associated with COVID-19 and non-COVID-19 mortality were examined using Cox proportional hazards models.

**Results:**

Between January 1, 2020, and December 31, 2021, a total of 6,616 deaths were recorded across the three HDSS sites, of which 5.2% were attributed to COVID-19. The COVID-19 mortality rate was highest in Matlab (58 deaths per 100,000 person-years), followed by Chakaria (15 deaths per 100,000 person-years) and the urban slums in Dhaka (11 deaths per 100,000 person-years). Household socio-economic status was significantly associated with COVID-19 mortality in the Matlab HDSS. Individuals from the lowest wealth tertile had 40% lower mortality compared to individuals from the highest wealth tertile (adjusted mortality rate ratio (aMRR): 0.60; 95% CI: 0.43–0.83). In contrast, no significant differences were observed for non-COVID-19 mortality across wealth tertiles. Age, sex, and marital status were significantly associated with both COVID-19 and non-COVID-19 deaths.

**Conclusion:**

Our data revealed that COVID-19 mortality was highest in the Matlab HDSS. Age, sex, and marital status were key determinants of both COVID-19 and non-COVID-19 mortality in Matlab. Notably, individuals from households in the lowest wealth tertile in Matlab had significantly lower COVID-19 mortality compared to those from households in the highest wealth tertile, while no wealth-related differences were observed for non-COVID-19 mortality.

**Supplementary Information:**

The online version contains supplementary material available at 10.1186/s12963-025-00448-z.

## Introduction

The COVID-19 pandemic has had a profound impact on populations worldwide, reshaping how people live, interact, and perceive health. Since its emergence in December 2019 till March 2025, COVID-19 has infected nearly 778 million people worldwide, caused 7.1 million deaths, and placed an unprecedented burden on healthcare systems and societies [1]. Of these global deaths, 808,871 occurred in the Southeast Asia region, including 29,499 deaths in Bangladesh [1].

In the pre-pandemic era, mortality in Bangladesh was primarily driven by non-communicable diseases (NCDs). In 2019, NCDs such as stroke, ischemic heart diseases, and chronic obstructive pulmonary diseases were responsible for most of the deaths in the country [2]. The COVID-19 pandemic thus presented a new challenge to the health system of Bangladesh, which was already struggling to cope with the double burden of non-communicable and communicable diseases.

Mortality is a key indicator of the impact of the COVID-19 pandemic, and mortality data are crucial for decision-making in public health [3]. Understanding mortality patterns and underlying factors influencing them is critical for evaluating pandemic response and strengthening preparedness for future crises. However, the number of reported deaths may not portray the true picture due to various factors, including systematic weaknesses in death reporting systems, particularly in countries with limited infrastructure [3], as well as challenges in assigning causes of death and accounting for the indirect effects of the pandemic on overall mortality [4, 5].

Across the globe, numerous national or sub-national studies have assessed the effect of the COVID-19 pandemic on all-cause mortality at various stages of the pandemic [6–8]. COVID-19 mortality rates are known to vary across demographic, socioeconomic, and geographical contexts, such as between rural and urban populations. This study utilised data from three Health and Demographic Surveillance System (HDSS) platforms in Bangladesh to examine mortality patterns and the influence of socio-demographic factors on mortality during the pandemic years (2020–2021), and to assess whether these factors differentially affected COVID-19 and non-COVID-19 deaths. In settings like Bangladesh, where the implementation and use of Civil Registration and Vital Statistics (CRVS) is weak [9], HDSS platforms provide a feasible alternative that enables longitudinal monitoring of defined populations and generating reliable estimates of births, deaths, migration, and fertility rates [10]. These systems, therefore, can offer critical insights into COVID-19 mortality in the absence of robust CRVS data.

## Materials and methods

### Study design

This study utilised data from three HDSS platforms run by the International Centre for Diarrhoeal Disease Research, Bangladesh (icddr, b) in three (Matlab, Chakaria, Dhaka) distinct socio-economic and environmental locations in Bangladesh, one of the most densely populated countries in the world. An HDSS is designed for longitudinal data collection on health, demographic, and socio-economic characteristics of a specific population cohort or community [11]. In these three platforms, each household is visited once every three months to record key demographic and health events like deaths, pregnancy outcomes, marriages, and migrations.

## Setting

**Fig. 1:**
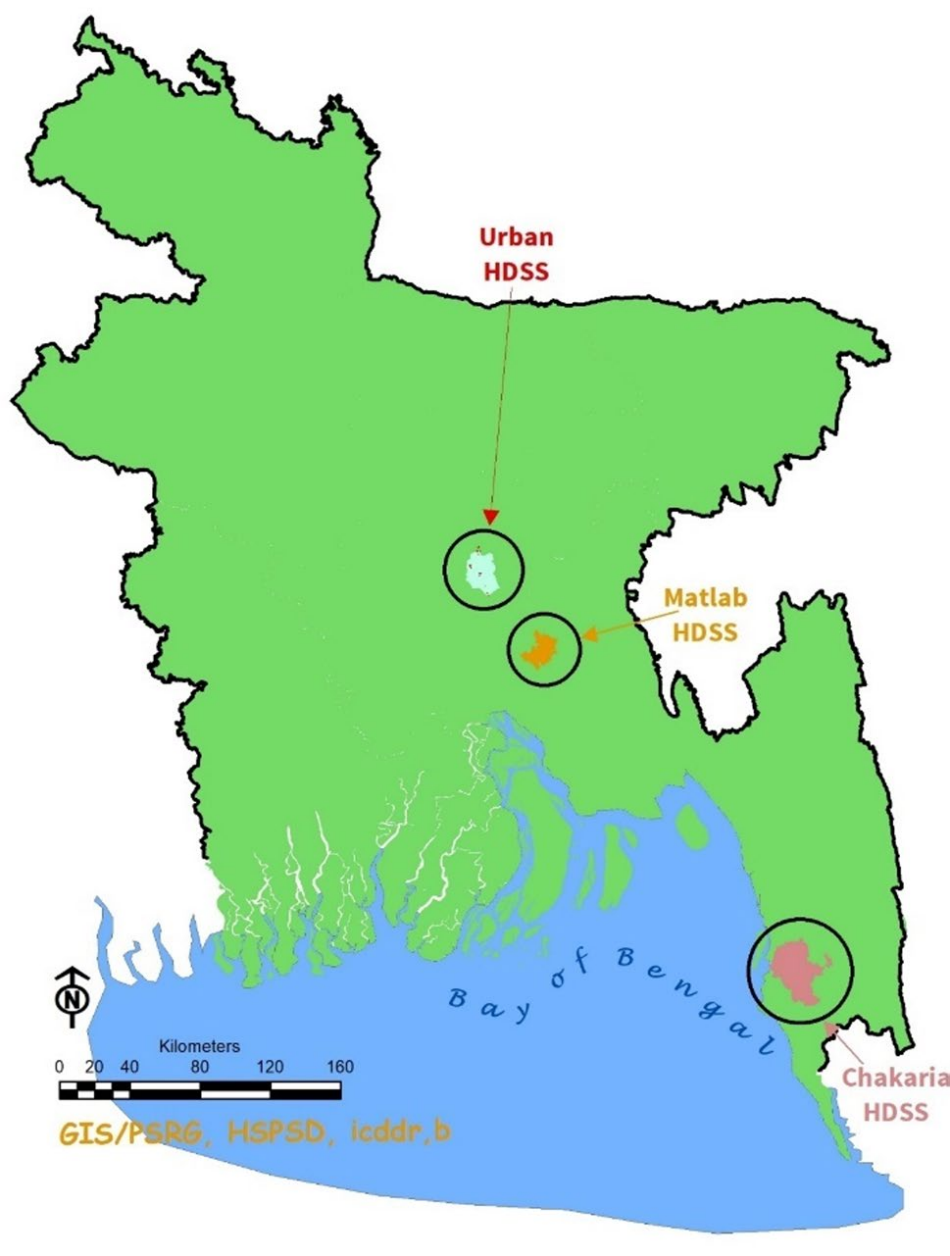
Geographic location of the Matlab, Chakaria, Urban HDSS sites in Bangladesh

The three HDSS sites are located in three geographic locations of Bangladesh, shown in Fig. 1. These HDSS sites (Matlab, Chakaria, and slums of Dhaka) represent different communities of Bangladesh (Table 1). The Matlab HDSS, established in 1966, represents a rural population and consists of about 240,804 people living in 142 villages in a rural district called Chandpur, which is about 55 km southeast of Dhaka [12]. The Chakaria HDSS, established in 1999, represents a coastal population. It covers 86,667 individuals living in 49 villages in a coastal sub-district called Chakaria, which lies within 7 km to 30 km of the coast of the Bay of Bengal [13, 14]. The Urban HDSS, started in 2016, covers five slums in Dhaka and adjoining urban areas and follows a population of 124,817 individuals [15, 16].

**Table 1:** Profile of icddr, b run HDSS sites in Bangladesh

Characteristics	Matlab HDSS	Chakaria HDSS	Urban HDSS
Established	1966	1999	2016
Population	240,804	86,667	124,817
Crude birth rate per 1000	22.0	24.2	16.6
Crude death rate per 1000	7.5	5.8	4.4
In-migration rate per 1000	46.0	24.6	159.5
Out-migration rate per 1000	52.8	34.1	149.8
Sex ratio (Male/Female)	82.2	98.2	97.2
Geographical location	Rural	Coastal	Urban Slum
Visiting cycle	Every 3 months	Every 3 months	Every 3 months
Data collection system	Electronic	Electronic	Electronic

Data Source: Annual scientific report of Matlab HDSS, 2019 [12]; Annual scientific report of Chakaria HDSS, 2019 [14]; Slum health in Bangladesh: insights from health and demographic surveillance, 2016 [16]

## Participants

**Fig. 2:**
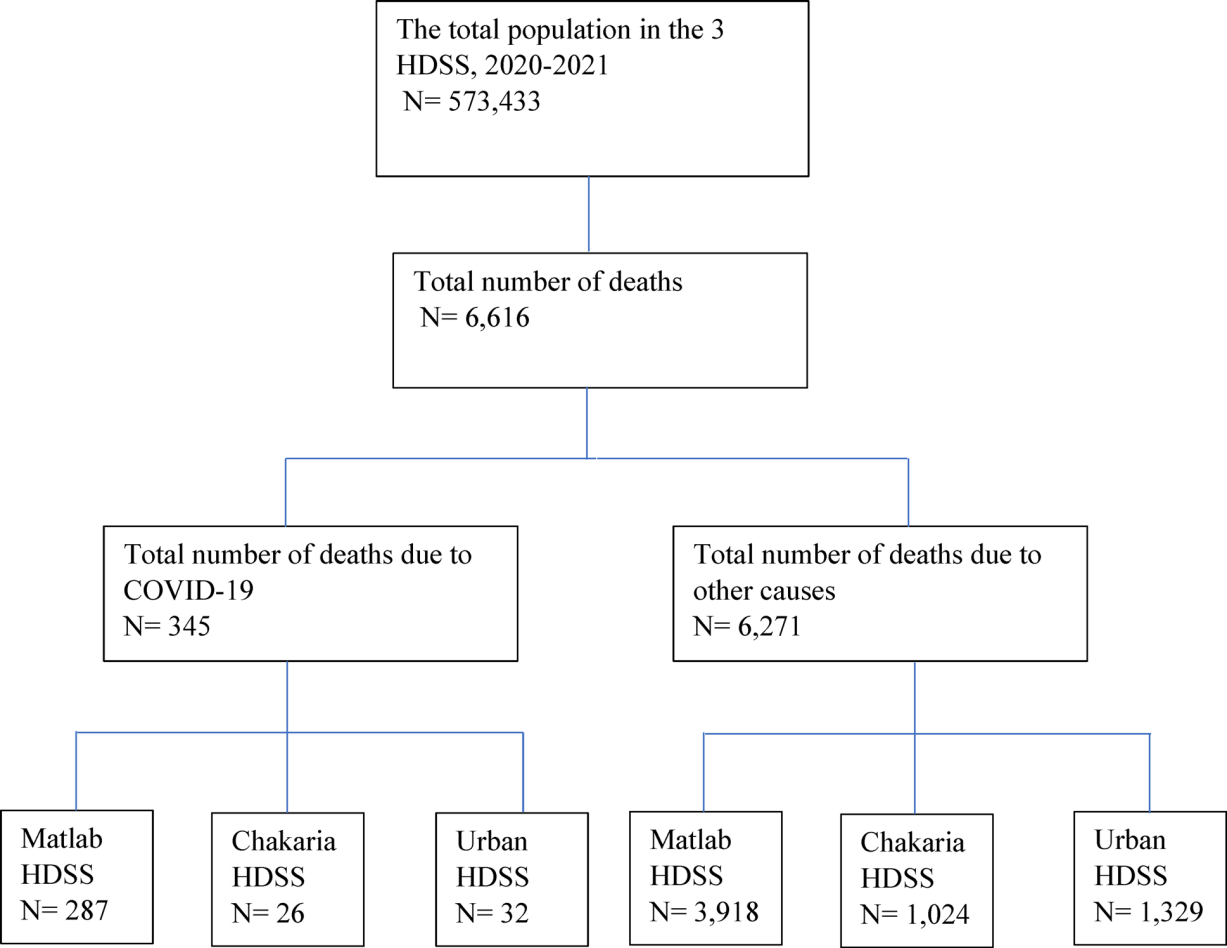
Study population

We included all individuals under the three HDSS areas and followed them up from January 1, 2020, to December 31, 2021 (Fig. 2). All households in Matlab, Chakaria, and Urban HDSS sites were visited quarterly by community health research workers (CHRWs), surveillance workers (SWs), and field workers (FWs), respectively, to enquire about demographic changes and health events. During the early part of the COVID-19 pandemic, between March 25 and November 9, 2020, CHRWs, SWs, and FWs collected data through mobile phones instead of in-person household visits as per the national precautionary guidelines [10]. Information from households that could not be reached at first contact was collected in subsequent rounds.

## Variables and measurement

Household socioeconomic status was assessed using data on household assets such as almirah, table, chair, choki/khat, showcase, electricity, television, by-cycle, refrigerator, watch/clock, sofa, fan, sewing-machine, and telephone. Principal component analysis was performed to produce a common factor score for each household. Household wealth tertiles were obtained by dividing the ascending factor scores into three equal categories, each comprising 33.33% of the households. All deaths between 2020 and 2021 were recorded.

## Verbal autopsy (VA) data

A web-based software application was designed and developed for the three HDSSs to facilitate the collection of VA data. After the CHRWs/SWs/FWs recorded a death, a trained field research supervisor (FRS) interviewed the close relatives or caregivers of the deceased after at least seven days of death, using the World Health Organization (WHO) 2016 VA instrument. The VA instrument is designed for use in settings where deaths occur outside the health sector and medical certification or cause of death is not available. In order to investigate COVID-19 mortality, the WHO updated the VA instrument with an additional set of questions for identifying deaths due to COVID-19. We used this updated version (v. 1.5.3) to conduct the verbal autopsy interviews [17]. Causes of death from the VA data were assigned by a medical personnel following the ICD-10 guidelines [18].

### Statistical methods

**Fig. 3:**
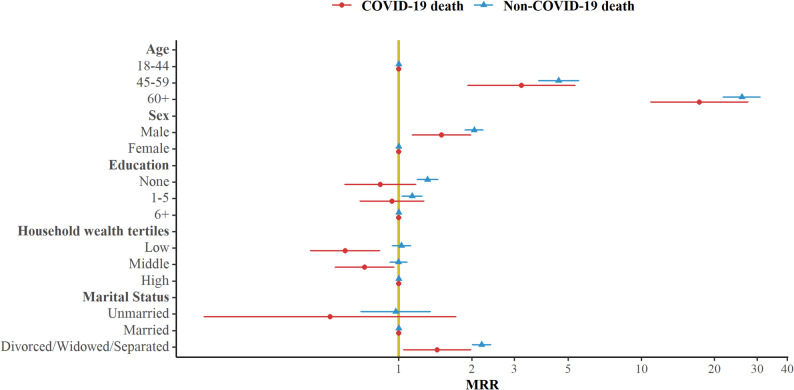
Forest plot showing the adjusted MRR for all factors in the Matlab HDSS site, Bangladesh, 2020–2021

Crude mortality rates were calculated as the number of deaths divided by person-years. Owing to the small number of COVID-19 deaths in Chakaria and Urban HDSS sites, analyses of factors associated with COVID-19 and non-COVID-19 mortality were restricted to the Matlab HDSS. Mortality rate ratios (MRR) with a 95% confidence interval were estimated using Cox proportional hazards regression models to assess associations between covariates and death. For Fig. 3 and the Supplementary Table, analyses were limited to the population aged 18 years and older. Age, sex, years of education, socio-economic status, and marital status were included as potential confounders in the adjusted models. Data analysis was conducted using STATA version 17 [19], and Fig. 3 was produced using the ggplot2 package in R.

## Results

A total of 573,433 individuals were followed across the three HDSS sites during 2020–2021. A total of 6,616 deaths were recorded over this study period, of which 345 deaths were attributed to COVID-19 and 6,271 to other causes. Among the COVID-19 deaths, 287 deaths were recorded in Matlab, 26 in Chakaria, and 32 in the Urban HDSS (Fig. 2). The proportion of COVID-19 deaths among all deaths was 7% in Matlab, 3% in Chakaria, and 2% in Urban HDSS.

**Table 2:** Background characteristics of the population in Matlab, Chakaria, and urban HDSS in Bangladesh, 2020–2021

Characteristics	Matlab (Rural)	Chakaria (Coastal)	Urban (Slum)	Total
	%			
Age				
<18 years	38.6	45.2	38.3	39.6
18–44 years	35.2	40.0	49.6	41.0
45–59 years	15.5	9.1	8.8	12.1
60 + years	10.7	5.7	3.3	7.3
Sex				
Male	46.1	49.0	49.1	47.6
Female	53.9	51.0	50.9	52.4
Education (years of schooling among people aged 5 years and above)				
None	20.4	20.6	32.4	24.7
1–5	23.7	35.1	40.1	31.3
6+	55.9	44.3	27.5	44.0
Marital status				
Unmarried	40.9	51.0	39.8	42.3
Married	51.8	44.7	55.1	51.7
Divorced/Widowed/Separated	7.3	4.3	5.1	6.0
N	276,539	97,689	199,205	573,433

Table 2 presents the background characteristics of the populations across the three HDSS sites. More than half of the total population was aged 18–59 years, while 39.6% were less than 18 years of age. The percentage of elderly individuals (aged 60 years and above) was highest in the Matlab HDSS (10.7%), followed by Chakaria (5.7%) and the Urban site (3.3%). In Chakaria, 45.2% of the population was less than 18 years of age, whereas nearly half of the population of the urban HDSS was within an economically productive age group of 18–44 years. Overall, more than half of the HDSS population were females (52.4%) and married (51.7%), while 6% were either divorced, separated, or widowed.

**Table 3:** COVID-19 and non-COVID-19 mortality rate (MR) across three HDSS sites in Bangladesh, 2020–2021

HDSS		Mortality rate per 100,000 person-years				
		COVID-19 deaths		Non-COVID-19 deaths		All deaths
Matlab		58 (287/494462)		792 (3918/494462)		850 (4205/494462)
Chakaria		15 (26/177358)		577 (1024/177358)		592 (1050/177358)
Urban		11 (32/292495)		454 (1329/292495)		465 (1361/292495)
Total		36 (345/964316)		650 (6271/964316)		686 (6616/964316)

Table 3 presents the mortality rates per 100,000 person-years for COVID-19 and non-COVID-19 deaths across the three HDSS sites. Mortality rates were highest in Matlab HDSS, while both Chakaria and Urban HDSS recorded comparatively lower rates.

Age was strongly associated with both COVID-19 and non-COVID-19 mortality in Matlab. Individuals aged 60 years and above had markedly higher mortality compared to those aged 18–44 years (aMRR: 17.34; 95% CI: 10.94–27.50 for COVID-19 and aMRR: 25.93; 95% CI: 21.73–30.94 for non-COVID-19 deaths) (Fig. 3). Compared with females, males had a 50% higher risk of COVID-19 mortality (aMRR: 1.50; 95% CI: 1.14–1.98) and twice the risk of non-COVID-19 mortality (aMRR: 2.04; 95% CI: 1.88–2.22). People belonging to the lowest wealth tertile had a 40% lower risk of COVID-19 mortality compared with people belonging to the highest wealth tertile group (aMRR: 0.60; 95% CI: 0.43–0.83). Divorced, widowed, or separated individuals had higher mortality rates compared to their married counterparts for both COVID-19 and non-COVID-19 death groups in Matlab. Further detailed results are shown in Supplementary Material.

## Discussion

Our study aimed to examine mortality during the COVID-19 pandemic years across three HDSS sites in Bangladesh and to identify contextual factors associated with COVID-19 and non-COVID-19 deaths. Age, gender, educational attainment, socio-economic status, and marital status are well-known socio-demographic factors associated with all-cause mortality [20–22]. Our analysis of MRRs in Matlab HDSS (Supplementary Material) similarly showed that aMRRs were higher among elderly individuals compared to younger individuals and among males compared to females for both COVID-19 and non-COVID-19 deaths. Compared to individuals from households in the highest wealth tertile, those from households in the lowest wealth tertile had a lower aMRR for COVID-19 mortality in Matlab HDSS.

The heightened vulnerability of older adults to COVID-19 is a global concern, evident in the consistently higher risk of mortality observed across diverse settings. Disruptions of primary healthcare services, coupled with inadequacies in the health system and a lack of elder-friendly facilities, contributed to further worsening of health outcomes among older adults during the pandemic [23]. A previous study conducted in Matlab reported 28% excess mortality among the elderly during the first phase of the pandemic [10]. Two HDSS-based studies conducted in Gambia and Kenya revealed very little or no excess mortality, but the Kenyan study reported significant excess mortality among the elderly (aged 65 years and above) [24, 25]. In our study, we found that very few COVID-19 deaths were recorded in the urban HDSS site, despite the well-known fact that densely packed informal settlements are conducive to the propagation of infectious agents like the COVID-19 virus. This can be due to the contrasting demographic profile of the Matlab and Urban HDSS populations. The proportion of the elderly in the rural Matlab HDSS is nearly three times that of the urban HDSS. Moreover, a 21-year follow-up of the Matlab HDSS population reported that young and healthy adults, particularly those without chronic morbidities, were more likely to migrate out of rural areas [26]. In Bangladesh, where nearly 68.5% of people live in rural areas [27], it is mostly the young and healthy adults who migrate to urban areas in search of better opportunities and frequently end up living in informal settlements. The younger age structure and relatively lower prevalence of comorbidities in urban areas may therefore partly be the reason behind the lower COVID-19 mortality observed in the urban HDSS population.

In contrast to much of the existing evidence, which indicates that COVID-19 mortality was higher among the poor segments of society [28], our study found that individuals in the highest wealth tertile had higher COVID-19 mortality compared to those in the lowest wealth tertile. In this context, evidence from the South Asian region is sparse [28]. A study by Middya and Roy in India reported a mixed picture where, in the COVID-19 death hotspots of Eastern and Western India, higher mortality was observed in more affluent areas [29].

An American study reported that in the early phase of the pandemic, COVID mortality was higher among the wealthier section of the county [30]. However, the trajectory reversed as the pandemic progressed further. In our study, individuals from households in the lowest wealth tertile had a lower aMRR for COVID-19 compared to individuals from households in the highest wealth tertile. The reasons for this are unclear and warrant further investigation. Understanding socio-economic differences and the practices and behaviors associated with them is critical for explaining disparities in health outcomes during pandemics. These insights can help decision-makers to optimise resource allocation, mobilization, and develop targeted interventions for specific groups. Overall, in our study, 5.2% of total deaths were attributed to COVID-19 using the VA tool. By comparison, a Kenyan study using VA and the COVID-19 Rapid Mortality Surveillance (CRMS) software attributed 1.8% deaths to COVID-19 [25].

The strength of our study is that it draws on direct observations from longitudinal population-based cohorts across three HDSS sites, enabling the analysis of mortality patterns with a high degree of completeness and timeliness. The use of standardized WHO verbal autopsy instruments ensures comparability and methodological rigor. By leveraging longitudinal HDSS data, our study offers rare insights into both COVID-19 and non-COVID-19 mortality in Bangladesh, particularly among vulnerable subgroups such as the elderly. These findings add to the limited evidence base from South Asia and can help inform evidence-based policies, targeted interventions, and future methodological improvements for pandemic-related mortality surveillance.

Our study also has a few limitations. All the VAs were conducted within 4 months of the death event. Although WHO hails VA as a standard tool for cause-specific mortality surveillance in resource-limited settings and recommends collecting information within 12 months of death [31], the possibility of recall bias cannot be completely ruled out. Furthermore, because the mortality data were derived from VAs, there may be some ambiguity in attributing deaths to COVID-19. While VA can provide valuable insights, it may not always accurately determine the precise cause of death and may have limitations in capturing complex realities [32].

Previous studies reported that populations from different races and ethnicities may communicate differently, which can influence outcomes, pointing to differences that may be more likely related to social structures than to biological and physiological factors [33–35]. In this study, we included only socio-demographic variables which are collected as part of routine data collection operations at the HDSS sites. There may be other potentially important factors, such as access to health care, cultural practices, and environmental conditions, that were not collected but may also influence mortality.

## Conclusion

This study provides robust, population-based evidence on COVID-19 and non-COVID-19 mortality from three HDSS sites in Bangladesh, offering rare insights from a low-resource setting with limited CRVS coverage. Our findings highlight the disproportionately high mortality among the elderly and men, as well as unexpected patterns across socio-economic strata, underscoring the need for more nuanced investigations into the social and contextual determinants of pandemic outcomes. By demonstrating the value of HDSS platforms and standardized VA tools for timely mortality surveillance, this work underscores their critical role in informing targeted, evidence-based interventions and improving preparedness for future public health emergencies, particularly in resource-limited settings where data gaps hinder accurate mortality estimates. Despite the uncertainty around the detection of COVID-19 deaths, the insights generated by this study contribute to the limited evidence base on the social and demographic determinants of COVID-19 and non-COVID-19 mortality in South Asia [28]. These findings can support the development of evidence-based policies and pave the way for more nuanced and robust methodological endeavors examining pandemic-related mortality in the future.

## Supplementary Information

Supplementary Material

## Data Availability

Data for computing mortality indicators for the three HDSS sites by year, age and sex are available from the MRC/Wits Agincourt Research Unit Data Repository on the following three links: https://data.agincourt.co.za/index.php/catalog/334, https://data.agincourt.co.za/index.php/catalog/335 and https://data.agincourt.co.za/index.php/catalog/336. Data on other covariates reported in this manuscript can be accessed through a formal request to the corresponding author.

## Abbreviations

HDSS: Health and Demographic Surveillance System
icddr,b: International Centre for Diarrhoeal Disease Research, Bangladesh
CRVS: Civil Registration and Vital Statistics
VA: Verbal Autopsy
ICD-10: International Classification of Diseases and Related Health Problems, tenth revision
MR: Mortality rate
MRR: Mortality rate ratio
aMRR: Adjusted mortality rate ratio
