# Supplementary material for: Patterns and determinants of COVID-19 mortality in Bangladesh: insights from three health and demographic surveillance systems across diverse socio-environmental settings
Source: Popul Health Metr. 2026 Jun 4;23(Suppl 2):78. doi: 10.1186/s12963-025-00448-z (PMC13235002; doi:10.1186/s12963-025-00448-z)
Supplement: Supplementary file 1 — Supplementary Material [file 12963_2025_448_MOESM1_ESM.docx]

Additional file 1. Mortality rates and mortality rate ratios of COVID-19 and non-COVID deaths in Matlab, Bangladesh, 2020-2021

| Factors | COVID-19 death | | |  | Non-COVID-19 death | | |
| --- | --- | --- | --- | --- | --- | --- | --- |
|  | MR/100,000 | Crude MRR | Adjusted MRR (95% CI) |  | MR/100,000 | Crude MRR | Adjusted MRR (95% CI) |
|  |  | (95% CI) |  |  |  | (95% CI) |  |
|  |  |  |  |  |  |  |  |
| Age |  |  |  |  |  |  |  |
| 18-44 | 17 (30/175268) | Ref. | Ref. |  | 114 (200/175440) | Ref. | Ref. |
| 45-59 | 59 (48/80963) | 3.44 (2.18-5.42) | 3.20 (1.93-5.31) |  | 638 (519/81408) | 5.58 (4.74-6.57) | 4.56 (3.77-5.50) |
| 60+ | 367 (208/56725) | 21.21 (14.47-31.10) | 17.34 (10.94-27.50) |  | 4762 (2818/59180) | 41.70 (36.13-48.13) | 25.93 (21.73-30.94) |
|  |  |  |  |  |  |  |  |
| Sex |  |  |  |  |  |  |  |
| Male | 112 (151/135250) | 1.47 (1.16-1.85) | 1.50 (1.14-1.98) |  | 1377 (1885/136878) | 1.49 (1.39-1.59) | 2.04 (1.88-2.22) |
| Female | 76 (135/177706) | Ref. | Ref. |  | 922 (1652/179150) | Ref. | Ref. |
|  |  |  |  |  |  |  |  |
| Education (years of schooling) |  |  |  |  |  |  |  |
| None | 133 (92/69250) | 2.03 (1.53-2.70) | 0.84 (0.60-1.17) |  | 2228 (1574/70656) | 4.11 (3.78-4.47) | 1.31 (1.19-1.45) |
| 1-5 | 104 (93/89045) | 1.59 (1.20-2.10) | 0.94 (0.70-1.27) |  | 1246 (1122/90016) | 2.30 (2.10-2.51) | 1.13 (1.03-1.24) |
| 6+ | 65 (101/154662) | Ref. | Ref. |  | 541 (841/155356) | Ref. | Ref. |
|  |  |  |  |  |  |  |  |
| Household wealth tertiles |  |  |  |  |  |  |  |
| Lowest | 63 (54/86038) | 0.49 (0.35-0.67) | 0.60 (0.43-0.83) |  | 1143 (994/86954) | 0.93 (0.86-1.02) | 1.03 (0.94-1.12) |
| Middle | 84 (81/96797) | 0.64 (0.49-0.85) | 0.72 (0.54-0.95) |  | 1164 (1138/97781) | 0.95 (0.88-1.03) | 0.99 (0.91-1.08) |
| Highest | 129 (135/104579) | Ref. | Ref. |  | 1219 (1288/105653) | Ref. | Ref. |
|  |  |  |  |  |  |  |  |
| Marital Status |  |  |  |  |  |  |  |
| Unmarried | 11 (3/28315) | 0.14 (0.04-0.43) | 0.52 (0.16-1.72) |  | 183 (52/28357) | 0.23 (0.18-0.30) | 0.97 (0.70-1.35) |
| Married | 77 (193/249198) | Ref. | Ref. |  | 797 (2000/250903) | Ref. | Ref. |
| Divorced/Widowed /Separated | 254 (90/35395) | 3.27 (2.55-4.20) | 1.44 (1.04-1.98) |  | 4044 (1485/36718) | 5.07 (4.74-5.42) | 2.19 (2.01-2.39) |
